# Supplementary material for: Effects of two different emotion-inducing methods on the emotional memory of non-clinically depressed individuals
Source: PLoS One. 2021 May 28;16(5):e0249863. doi: 10.1371/journal.pone.0249863 (PMC8162709; doi:10.1371/journal.pone.0249863)
Supplement: Attachment — Submitted filename: mo_review.docx [file pone.0249863.s002.docx]

Lin et al. investigated the impact of emotional induction on memory for faces in normal depressed participants, addressing the question whether a depressed profile is accompanied by heightened sensitivity for negative memories. Two facial recognition experiments were conducted, which differed according to the emotion-induction method employed. Experiment 1 used faces with neutral expressions that, during a training phase, were associated with negative, positive or neutral feedback and then tested recognition memory for these faces. Experiment 2 exposed participants to faces with negative, positive or neutral expressions during the training phase and evaluated whether recognition memory was affected by the intrinsic emotional quality of the faces. Very different results were obtained in the two experiments. In the first, normal participants show better memory for positive as compared to negative faces but depressed participants deviated from this pattern by not showing this “suppression” of memory for negative faces. In contrast, in Experiment 2 recognition memory was better for previously seen faces with negative as compared to positive and neutral expressions, and there were no differences between depressed and non-depressed participants, suggesting that memory performance on this task variant is enhanced by the emotional saliency/arousal value of the face stimuli and this, to the same extent in the two groups.

This study has merits from both the methodological and theoretical point of views and could make a useful contribution to the field. The testing procedures and statistics applied appear appropriate. However, several points need to be addressed/clarified and the text requires some improvements.

My comments are listed below.

1) What is the relevance of non-clinical depressed subjects for study aimed at understand cognitive/emotional processing in depression? Is there any evidence ofa continuum between non-clinical and clinical depression? That high BDI scores in non-clinical populations are a predictor of risk for depression? Since, in several parts of the paper, participants as referred to as “healthy” and “depressed”, it is important point to address this point, at least as a cautionary note in the interpretation of the data.

2) p.9 2^nd^ par (“Stimuli”), line 2 : “All facial pictures in the system were obtained by all 60 participants” Unclear, please rephrase.

3) p.9 2^nd^ par (“Stimuli”) : Who performed the valence rating of the pictures? The same 60 subjects that participated in the memory experiment or a separate group? If they are the same, when did they perform the rating?

4) p.10 1^st^ par, line 2: Judging the “identity” is not exactly what participants were asked to do. It seems more like a personality or trustworthiness judgement was requested.

5) p.10 1^st^ par, line 4: “The participants pressed a key to give feedback”. I think ‘to receive feedback’ is meant here, right? Also, as I understand it, on each face pair presentation, there were three possible options to choose from: left is a cheater, right is a cheater, none is a cheater. How did participants express their choice? Vocally? By pressing different keys?

6) p.10 1^st^ par: As I understand it, although I’m not 100 percent sure, the two simultaneously presented training faces were tagged as negative following a “wrong” feedback (or as positive following a “correct” feedback) and both were used in the recognition test. Is that correct?

7) p.10 2^nd^ par, line 2: “The face persisted until the reaction disappeared” Unclear. What do the authors means? That the face remained displayed until the participant responded? Please re-phrase more clearly.

8) p.10 4^th^ par (Results): Several comparisons are made between healthy and “depression”. It should probably read “depressed”

9) p.12 4^th^ par (Stimuli): The authors present the results of an analysis confirming the different valences of the test stimuli, but do not explain how the data were obtained.

10) p.13 2^nd^ par: Here again, the reader is left to guess what the participants were instructed to do during the learning phase. Please be more explicit.

11) p.14 2^nd^ par, 1^st^ line: “The second experiment was”: I think the authors meant ‘The *aim of* the second experiment was’.

12) p.15 1^st^ par, line6-7: I am skeptical about the interpretation of higher RTs in depressed participants: it is not obvious why, when the task is not to identify not the emotion, but to recall the familiarity of a face, a tendency to misinterpret emotions should slow down response times. Furthermore, if a bias to assign a negative emotion to neutral face did contribute to slow down RTs, why are these results opposite to those obtained in Experiment 1 with neutral faces only (i.e. faster RTs in depressed participants)? Actually, the opposite direction of the RT difference between normal and depressed participants between the two experiments is quite puzzling.

13) p.15 2^nd^ par, line 3-4: “on the performance of memory”: ‘on memory performance’ wound read better.

14) p.16 1^st^ par line 7: “individuals with depression have a stronger attention bias toward negative materials and the further promotion of subsequent memory effects” The second part of this sentence is unclear. Please rephrase.
